# Close of Volume Seven

**Published:** 1872-01

**Authors:** 


The Bistoury.

ELMIRA, N. Y., JANUARY, 1872.

The BISTOURY is published Quarterly, upon the 1st of April, July, October and January, at Fifty Cents a year, in advance.

### CLOSE OF VOLUME SEVEN.

This number closes Volume Seven. It is now three years since we began the publication of the BISTOURY in magazine form, giving us three hundred and fifteen pages of matter, which, for real, solid merit and usefulness to the heads of families, and to the general practitioner of medicine, is unsurpassed by any journal, of whatever character, of which we have any knowledge. It will be seen that we have carefully indexed the contents of the journal for the past three years, and have arranged alphabetically the subjects treated upon, both in the Domestic Medical Department, and in the Medical Items and News; so that the mother can quickly refer to the BISTOURY for a remedy for any simple ailment of a member of her household, while the physician can in like manner, consult the most eminent authorities of the world upon the treatment of almost any disease known to man.

The Domestic Medicine contains one hundred and forty-two articles upon health matters, and useful recipes for the family, while in the Medical Items and News we find three hundred and eighty-seven formulæ of immense value to every intelligent physician. These formulæ alone, are worth many times more than the subscription price of our journal, but only cost the small sum of $1.50, or three years' subscription to the BISTOURY. We would have our professional readers look over the contents of MEDICAL ITEMS AND NEWS, and see what an immense amount of useful matter we have prepared for them during the past three years.

With the April number begins a new volume, and with it we will make an important change in the paging of the BISTOURY. We intend having the running page of the journal at the bottom, instead of at the top, as usual; while the MEDICAL ITEMS AND NEWS will be paged at the top, keeping the paging of this department separate and distinct from the other portion of the magazine; and at the expiration of three years we will publish a separate and complete index and title page for it, when the physician can separate it from the balance of the matter and have it bound, giving him an elegant volume, every three years, on everything that is new and valuable in the treatment of disease. We shall also devote more time to the preparation of this department, hoping that our professional brethren will approve of our plan, and lend us their help by subscribing liberally.

Our Domestic Medicine Department will be continued and enlarged, under the new heading of

INFORMATION FOR THE HOUSEHOLD, in which will be given simple and effective recipes for the treatment of ordinary diseases, in the absence of the regular medical adviser, together with recipes that will be found useful and welcome in every family.

Our contributors, Mr. Ben. H. Pratt, Rev. Thos. K. Beecher, Dr. Hayes and Uncle Bob, who have so pleased and instructed our readers during the past year, will continue with us during the new volume; when we expect also to introduce some new names who have engaged to write for us, so enlarging our list of contributors.

Quacks, Humbugs and Swindlers will receive "particular fits," as usual—threats of violence and prosecution will avail them nothing; we will treat them just as we have heretofore—unmercifully.

Therefore, look out for the April number, and send in your orders early. We enclose the usual envelope and blank, by which means subscriptions can be sent us.

Many who took the BISTOURY when it was a *free* journal, have continued to take it since fifty cents has been asked for it, without paying up. They will have an opportunity of doing so now, voluntarily, or be compelled to when the arrearages amount to enough to warrant the sending of an agent for its collection.

We call attention to our premium list, and invite our friends to renew their subscriptions, and, if our inducements are sufficient, to send us a list of names from their respective localities, for our journal. We ask no one to work for nothing, but offer large *cash* premiums for every subscriber.

---

THE son of Dr. Jenner, and nephew of the discoverer of vaccination, is now, through adverse circumstances, living in a very small cottage, with hardly the necessaries of life.

